# Prevalence, awareness, treatment, and control of hypertension in southwestern China

**DOI:** 10.1038/s41598-019-55438-7

**Published:** 2019-12-13

**Authors:** Xiao-Bo Huang, Yang Zhang, Tzung-Dau Wang, Jian-Xiong Liu, Yan-Jing Yi, Ya Liu, Rong-Hua Xu, Yong-Mei Hu, Mao Chen

**Affiliations:** 1 Department of Cardiology, the second people’s hospital of Chengdu, Chengdu, Sichuan China; 2 Department of Cardiology, West China Hospital, Sichuan University, Chengdu, Sichuan China; 3 Division of Cardiology, Department of Internal Medicine, National Taiwan University Hospital, Taipei City, Taiwan; 4 Department of Geriatrics, The second people’s hospital of Chengdu, Chengdu, Sichuan Province China; 5 Stroke Center, the second people’s hospital of Chengdu, Chengdu, Sichuan Province China

**Keywords:** Epidemiology, Epidemiology, Epidemiology, Epidemiology, Risk factors

## Abstract

This study investigated the prevalence, awareness, treatment, and control of hypertension and associated factors among urban adults in southwestern China. The study was conducted from 2013–2014 and used a multistage cluster sampling method to select a representative sample of 11,517 people in southwestern China, aged 35–79 years. Hypertension was defined as either systolic blood pressure of 140 mmHg or greater, diastolic blood pressure of 90 mm Hg or greater, or self-reported current treatment for hypertension with antihypertensive medications. In the study population, hypertension prevalence was found to be 38.4%, with rates of 40.0% and 37.5% for men and women, respectively (*p* = 0.03). Hypertension prevalence increased with age in both men and women (trend *p* both <0.01). Among hypertensive patients, 47.9% were aware of their hypertension, 40.1% were undergoing antihypertensive treatment, and 10.3% achieved BP control. A multiple-factor analysis revealed that age, male gender, low educational achievement, family history of hypertension, overweight or obesity, abdominal obesity, and hypertriglyceridemia were positively related to hypertension, while physical exercise was negatively related to hypertension. The prevalence of hypertension among urban adults aged 35 to 79 years in southwestern China was high, while levels of awareness, treatment, and control of hypertension were low. Multifaceted interventional measures are needed to solve the unmet needs.

## Introduction

Hypertension is an important public-health challenge worldwide and a major risk factor leading to stroke, myocardial infarction, heart failure, renal failure, and ultimately death^1,2^. Socioeconomic and demographic transitions occurring in many developing countries have contributed to the burden of hypertension^3–5^. With China’s rapid economic development, the disease burden has changed from communicable diseases to non-communicable diseases, and hypertension has become the leading cause of death among Chinese adults^6^.

In southwestern China, a population of 200 million people inhabit over 2,300,000 square kilometers^7^, almost covering a quarter of China’s territory. Once an underdeveloped region, it has benefited from the implementation of a development strategy in western China in the year 2000, which has resulted in significant economic and infrastructural growth in southwestern China, making the region the fastest growing area in the country^8^. According to data released in 2016 from the National Bureau of Statistics, Chengdu and Chongqing, the two central cities in southwestern China, had GDPs of 1,217 billion and 1,774.1 billion, respectively, compared to 275 billion and 390.7 billion, respectively, in 2006. This rapid economic development has led to lifestyle changes, including increased unhealthy nutrition, tobacco consumption, and reduced physical activity, which in turn, have led to increased prevalence of hypertension and other cardiovascular diseases, such as high blood pressure^9,10^.However, epidemiological investigations of hypertension in the southwestern region are lacking, with one surveyed conducted by Liu *et al*. in Chongqing^11^. Thus, the current study assessed the prevalence of hypertension among urban adults aged 35 to 79 years in Chengdu and Chongqing to provide valuable information to aid prevention and treatment of hypertension.

## Results

**Figure 1:**
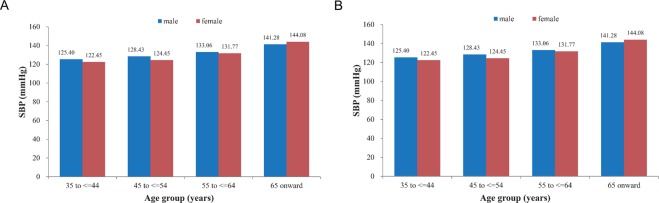
Association among age, gender, and blood pressure. Systolic blood pressure (SBP) increased with age in both sexes (**A**, Trend analysis: Male: P < 0.001; Female: P < 0.001) but diastolic blood pressure (DBP) did not (**B**, Trend analysis: Male: P > 0.05; Female: P > 0.05).

**Figure 2:**
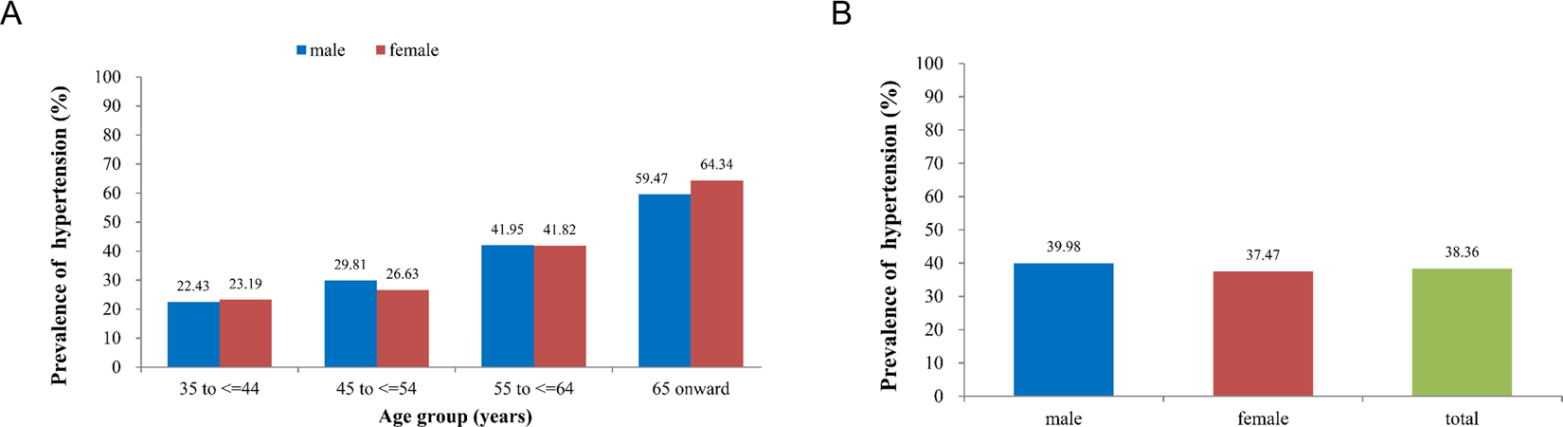
Association among age, gender, and hypertension. (**A**) Prevalence of hypertension between different sexes. Test of prevalence of hypertension between male and female: P = 0.03. (**B**) The prevalence of hypertension increased with increasing age in both sexes. Trend analysis: Male: P < 0.001; Female: P < 0.001.

The basic characteristics of the study population are shown in Table 1. Of the 11,517 participants, 4,809 were males and 7,430 were females, with a mean age of 55.1 ± 11.0 years and with males having a higher mean age than females. In terms of education level, 76.5% of the participants’ did not have high school degrees, and men had higher education levels than women (*p* < 0.001). However, compared to women, men had higher values for WC, SBP, and DBP, had higher rates of drinking and smoking, and had higher personal monthly incomes (all *p* < 0.001). Women had higher BMIs, pulse rates, TC, 2hPG, and were more likely to exercise regularly (all *p* < 0.05). There were no differences in TG, FPG, or family history of hypertension. SBP increased with age (*p* < 0.01) in both sexes (Fig. 1A) but DBP did not (*p* > 0.05, Fig. 1B). As shown in Fig. 2A, approximately 38.4% of all participants had hypertension (4,418/11,517), with prevalence rate of 40% among men, compared to 37.5% among women (*p* = 0.03). The prevalence of hypertension increased with increasing age (*p* < 0.01) in both sexes (Fig. 2B).

**Table 1:** General characteristics of the study population.

Variables	Total (*n* = 11517)	Male (*n* = 4087)	Female (*n* = 7430)	*P*
Age (years old), mean (SD)	55.1 (11.0)	56.4 (11.2)	54.4 (10.8)	<0.001
Current smoking (%)	2336 (20.3)	2039 (49.9)	297 (4.0)	<0.001
Current drinking (%)	1834 (15.9)	1556 (38.1)	278 (3.7)	<0.001
Below high school education degree (%)	8809 (76.5)	2765 (67.7)	6044 (81.4)	<0.001
Personal monthly income (<2000 yuan) (%)	9372 (81.4)	3128 (76.5)	6244 (84.0)	<0.001
Physical exercise (%)	6802 (59.1)	2339 (57.2)	4463 (60.1)	0.012
Family history of hypertension (%)	2444 (21.2)	866 (21.2)	1578 (21.2)	0.998
BMI (kg/m^2^), mean (SD)	24.1 (6.8)	23.8 (6.3)	24.2(7.0)	<0.001
WC (cm), mean (SD)	81.7 (26.4)	83.5 (28.8)	80.8 (24.9)	<0.001
SBP (mmHg), mean (SD)	130.9 (21.4)	132.7 (20.4)	129.9 (21.9)	<0.001
DBP (mmHg), mean (SD)	81.2 (21.2)	82.3 (17.1)	80.6 (23.1)	<0.001
Pulse rate (beats/min), mean (SD)	80.0 (25.1)	79.1 (26.7)	80.6 (24.1)	<0.001
TC (mmol/L), mean (SD)	4.6 (0.9)	4.5 (0.9)	4.7 (0.9)	<0.001
TG (mmol/L), mean (SD)	1.6 (1.3)	1.6(1.2)	1.6 (1.3)	0.217
FPG (mmol/l), mean (SD)	5.7 (2.0)	5.7 (1.9)	5.7 (2.1)	0.709

The awareness, treatment, and control of hypertension in the different groups are shown in Table 2. Among all hypertensive participants, the awareness and treatment rates were higher among women compared to men (50.5% versus 43.5%, *p* < 0.001 and 43.0% versus 35.3%, *p* < 0.001, respectively). Among those who were aware they had hypertension, there were no statistically significant differences in treatment between females and males (85.1% versus 81.3%, *p* = 0.08). Among all hypertensive participants, the control rate had no statistically significant difference between women and men (11.1% versus 8.9%, *p* = 0.06). For those who had been treated for hypertension previously, the control rate was 25.1% among men and 25.8% among women, with no significant differences (*p* = 0.95).

**Table 2:** Awareness, treatment, and control among urban hypertensive adultsaged 35 years or older in southwest China (%).

	Total	Male	Female	*P*
Awareness	47.9 (46.4–49.4)	43.5 (41.1–45.9)	50.5 (48.7–52.4)	<0.001
**Treatment**				
among all with hypertension	40.1 (38.7–41.6)	35.3 (33.0–37.6)	43.0 (41.1–44.8)	<0.001
among those who were aware of their hypertensive conditions	83.8 (82.2–85.4)	81.3 (78.4–84.1)	85.1 (83.2–86.9)	0.08
**Control**				
among all with hypertension	10.3 (9.4–11.2)	8.9 (7.5–10.3)	11.1 (9.9–12.3)	0.06
among those treated	25.6 (23.6–27.6)	25.1 (21.6–28.7)	25.8 (23.4–28.3)	0.95

A multivariate logistic regression analysis was performed to identify significant determinants of hypertension, and the results are shown in Table 3. The results indicated that age, male gender, family history of hypertension, overweight or obesity, abdominal obesity, and TC were positively associated with hypertension, and educational level of high school or above and physical exercise were negatively associated with hypertension (all *p* < 0.05).

**Table 3:** Multivariable-adjusted ORs and 95% CI for hypertension among the adults aged 35 years or older in southwest china.

Variable	Odds Ratio (95% CI)	P Value
male	1.40 (1.26–1.56)	<0.001
**Age group (years) (ref:** ≥**35 –** ≤ **44)**		
≥45 – ≤ 54	1.27 (1.12–1.45)	<0.001
≥55 – ≤ 64	2.14 (1.90–2.42)	<0.001
≥65 – ≤ 79	4.33 (3.76–4.99)	<0.001
below high school education degree	1.40 (1.26–1.57)	<0.001
Family history of hypertension	1.48 (1.34–1.64)	<0.001
physical exercises	0.62(0.56–0.68)	<0.001
BMI ≥ 25 kg/m^2^	1.72 (1.56–1.91)	<0.001
Waist circumstance (male: ≥90 cm, female: ≥85 cm)	1.45 (1.31–1.61)	<0.001
TG ≥ 1.7 mmol/L	1.28 (1.17–1.40)	<0.001

## Discussion

This study assessed the prevalence of and factors related to hypertension among urban adults aged 35 to 79 years in Chengdu and Chongqing, from September 2013 to March 2014. Overall, the prevalence of hypertension was 38.4% and rates of awareness, treatment, and control of hypertension among the study population were 47.9%, 40.1%, and 10.3%, respectively. Compared to previous epidemiological data of hypertension in this region, the prevalence rate (38.4%) was significantly higher than the prevalence rate of hypertension (23.0%, 565/2459) among 35–79 year old individuals in urban communities in Chengdu investigated in 2002^12^ and the prevalence rate of hypertension (31.3%, 1040/3325) among 35–79 year-old individuals in urban communities in Chongqing studied in 2004^13^. In the past 10 years, due to rapid economic development, the prevalence rate of hypertension in southwestern China has also risen rapidly. This and many previous studies have shown that overweight and obesity are important risk factors for hypertension^11,14–16^. The development of the economy has led to a great increase in unhealthy lifestyles, such as binge eating and drinking and lack of exercise, as well as a significant increase in obesity, which has resulted in a significant rise in the prevalence rate of hypertension. It is of great significance for community residents to adopt healthy lifestyles to control weight and waist circumference, which may effectively curb the rise in the prevalence rate of hypertension.

Data from the China National Nutrition and Health Survey in 2002 showed a prevalence rate of 18%^17^; however, data from the China Patient-Centered Evaluative Assessment of Cardiac Events Million Persons Project, conducted between 2014 and 2017, showed a prevalence rate of 44.7%^14^. The prevalence rate of hypertension in southwestern China from 2013–2014 was higher than that in the country 10 years prior and lower than the country-wide prevalence rate determined during the same period of study. This investigate also revealed that male gender, low levels of education, family history of hypertension, hypertriglyceridemia, and abdominal obesity were risk factors for hypertension, which is consistent with previous studies^14,18,19^.

The prevalence of hypertension was higher among men compared to women (40.0% vs. 37.4%, *p* = 0.03), which was also consistent with previous research^18,20,21^. With increasing age, levels of SBP and DBP and the prevalence of hypertension increased gradually in both men and women. More than a third of individuals between 55 and 65 years of age and more than half of individuals older than 65 had hypertension in this study. Therefore, for these populations, monitoring blood pressure once every 3–6 months is recommended. Family history of hypertension is an independent risk factor for hypertension; thus, people with a family history of hypertension require more frequent monitoring of blood pressures.

Among individuals with hypertension, the awareness, treatment, and control rates were 47.9%, 40.1%, and 10.3%, respectively. The awareness rate of hypertension (47.9%) among adults aged 35–79 years in southwestern China was similar to the national rate (44.7%) from 2014–2017, while the treatment (40.1%) and control (10.3%) rates were both higher than the national treatment (30.1%) and control (7.2%) rates^14^. This could be due to the fact that the current investigation was conducted in urban areas of Chengdu and Chongqing with relatively developed economies, higher levels of education, better community medical equipment, and relatively high levels of diagnosis and treatment for hypertension compared to national averages.

The awareness rate of hypertension among all hypertensive participants were higher among females compared to males (50.5% versus 43.5%, p < 0.001), which showed that females were more likely to monitor blood pressures. While half of the women included in this study were not aware that they had hypertension, and more than half of men did not realize they had hypertension, indicating that enhanced blood pressure monitoring is required for these individuals. Among those who were aware they had hypertension, the treatment rate was 85.1% in females and 81.3% in males, which showed that most people who were aware they had hypertension had recognized the necessity of hypertension treatment. However, the treatment rate of 40.2% is low, and more than half of the hypertensive patients were not undergoing antihypertensive treatment, possibly due to the low rate of hypertension awareness, and enhance blood pressure monitoring may also improve the treatment rate. Among hypertensive patients undergoing treatment, the control rate was 25.8% among females and 25.1% among males, and overall, only 1/10 of all hypertensive patients had their hypertension controlled; in comparison, the control rate is 1/5 and 1/6 in the United States and Canada, respectively^22^. Stroke is the leading cause of death and adult disability in China^9,23^, and previous studies have shown that hypertension is an independent risk factor for stroke^24,25^. Blood pressure above 115/75 mmHg, with differences of 20 mm Hg in SBP (approximately equivalently to 10 mm Hg in DBP) are associated with more than two fold differences in stroke and ischemic heart disease death rates^26^. Rapidly increased prevalence in hypertension and an extremely low control rate might lead to high incidence of stroke and other cardiovascular diseases, and without improvements, China will experience an outbreak of cardiovascular diseases over the next decade.

Significant efforts have been made by the Chinese government to improve the present situation, including promoting the development of basic medical centers and trained general medical practitioners and implementing a basic health insurance scheme to cover all residents. An essential drug system has also been implemented lower the price of antihypertensive drugs so that more people can afford treatment^11^. However, the gap between China’s prevalence, awareness, and control of hypertension and those of developed countries remains, and during the past decade, increasing prevalence of overweight and obesity, poor compliance with medication regimes, and lack of health education significantly affect hypertension control among low-income populations^27,28^. For example, in the 1980s, Canada’s hypertension control rate was 13%^27,28^; however, after two decades of effort the control rate was 65% in 2009^22^. Thus, China must do more to decrease the high prevalence of hypertension and to improve low awareness and low control rates among the population. Previous studies have recommended effective actions for controlling hypertension, such as restricting unhealthy food; supporting physical activity; increasing overall government spending on hypertension; promoting free blood pressure screening and public awareness programs; integrating hypertension management into routine primary care practices; promoting structured physician education programs to reduce clinical inertia and to improve guideline adherence; and providing universal access to affordable, high-quality, and effective antihypertensive drugs^22^.

The current study does have some limitations. First, as a cross-sectional study, the findings cannot be used to establish a conclusive cause-and-effect relationship between risk factors and hypertension. Second, the study was conducted in urban areas of Chengdu and Chongqing and did not include rural and small cities; thus, the results may not be representative of the prevalence of hypertension among small cities and rural residents in southwestern China.

## Conclusions

The prevalence of hypertension among urban adults aged 35 to 79 years in southwestern China is high and has increased rapidly, while rates of awareness, treatment, and control of hypertension remain low. Improved hypertension-related health knowledge should be delivered to improve public awareness of the disease and to strengthen the capacities of community health services to manage it.

## Materials and Methods

### Ethics statement

The experimental study involving human subjects was in accord with the Helsinki Declaration. And all experiments in this study were approved by the ethics committee of Second People’s Hospital of Chengdu, China. The informed consents were obtained from all subjects.

### Study population

From September 2013 to March 2014, multistage, stratified sampling was conducted among people aged 35 to 79 years who lived in urban communities within Chengdu and Chongqing, using both a questionnaire and physical measurements. During the first phase of this study, the Jinjiang, Longquan, and Chenghua districts were randomly selected from the urban area of Chengdu, and the Yubei and Jiangbei districts were randomly selected for Chongqing. During the second phase, a random subdistrict was selected from each major district, and during the third stage, one community was randomly selected from each subdistrict, resulting in a sample consisting of five random communities.

### Inclusion and exclusion criteria

Residents aged 35–79 years who had lived in the selected communities for more than five years were included in the study. People with histories of secondary hypertension, mental illness, malignant tumors, renal failure requiring dialysis, or who refused to participate in the inquiry were excluded. From September 2013 to March 2014, 13,378 people were invited to participate. Due to missing demographic information and weight, blood pressure, WC, or body mass index (BMI) data, 1,861 patients were excluded. Thus, 11,517 patients were included in the final analysis.

### Data collection

More than 30 investigators were trained for data collection. All subjects filled out the same onsite questionnaire, according to the cardiovascular survey methods set out by the World Health Organization^29^, which included demographic characteristics; lifestyle risk factors; personal and family histories; height, weight, WC, and blood pressure measurements;level of awareness of hypertension; and type of treatment. The questionnaire also included fasting blood-glucose, triglycerides (TG), and total cholesterol (TC) levels. BMI was calculated as weight (kg) divided by height (meters) squared, and when measuring height and weight, subjects were required to be barefoot and to be wearing only lightweight clothing. Investigators measured the minimum circumference between the inferior margin of the ribcage and the crest of the iliac to obtain WC measurements^30^. Thirty minutes before measurement were taken, subjects were told not to drink coffee, tea, or alcohol and to refrain from smoking or exercising. The subjects took a five-minute seated rest, then standardized mercury sphygmomanometers were used to measure their sitting blood pressures. Systolic blood pressure (SBP) and diastolic blood pressure (DBP) were recorded at first appearance (phase I) and at disappearance (phase V) of Korotkoff sounds, and two blood pressure readings were obtained and averaged.

### Diagnostic standards

According to the United States’ JNC-8 standards, high blood pressure was defined as an SBP ≥ 140 mm Hg and/or a DBP ≥ 90 mm Hg and/or a diagnosis of hypertension currently treated by antihypertensive drugs^1^. For this study, awareness of hypertension was defined as self-reporting of any previous diagnosis of hypertension by a healthcare professional. Treatment of hypertension was defined as the use of a prescription medication for management of high BP at the time of the interview. Control of hypertension was defined as pharmacological treatment of hypertension associated with an average SBP < 140 mm Hg and an average DBP < 90 mm Hg during the study visit^1^. Overweight was defined as a BMI of 25.0–29.9 kg/m^2^, and obesity was defined as a BMI of 30.0 kg/m^2^ or more^31^. Central obesity was defined as a WC of 90 cm or more in men and of 85 cm or more in women^32,33^. Hypertriglyceridemia was defined as a TG level ≥ 1.7 mmol/L, and hypercholesterolemia was defined as a TC level ≥ 5.7 mmol/L, based on the criteria of the NCEP Adult Treatment Panel III Report^34^. A history of smoking was defined as smoking at least once per day for more than a year, and currently having smoked or quit smoking for less than three years. A history of drinking was defined as drinking at least once a week over a year, and currently having drank or quit drinking for less than three years. Family history of hypertension was defined as immediate family members having hypertension, and physical exercise was defined as performing at least one exercise session per week. Higher education level was defined as the educational degree of high school or higher.

### Statistical analysis

EpiData 3.02 database software was used to record data from the questionnaires. Data input was completed by two researchers, who also performed data checking and correction. Categorical variables were presented as frequency (percentage), and Chi-square or Fisher exact tests were used for inter-group comparisons. For continuous variables, mean ± SD was used to represent data, and a two-sample t-test was used for inter-group comparisons. Frequency and 95% confidence interval were used to describe awareness of, treatment of, and control rates for hypertension, and Chi-square or Fisher exact tests were used for inter-group comparisons. A multivariate logistic regression model was used to estimate the odds ratios and corresponding 95% confidence intervals to investigate the significant risk factors of hypertension. A duplex bar chart was used to describe trends in SBP, DBP, and prevalence of hypertension among age groups, and a Cochran-Armitage test and generalized linear model were used to test trends in hypertension prevalence and SBP and DBP, respectively. A bar chart was used to describe the prevalence of hypertension among male, female, and total populations. SAS software was used to conduct statistical description tests to obtain differences and to perform a multivariate analysis.
